# Death’s Doings in England

**Published:** 1878-03

**Authors:** 


**DEATH'S DOINGS IN ENGLAND.**—Half of all who died in England and Wales during 1858, were under seventeen years of age. More than one hundred thousand died of disease of the lungs. Consumption killed twenty-five thousand, Pneumonia killed more than consumption, and Bronchitis killed more than Pneumonia. The common name of the last-mentioned is "Inflammation of the lungs," and is usually fatal in a few days. In cases of recovery, it is painfully slow, sometimes requiring weary years. It is always caused by the application of cold, and uniformly, in one of two ways, either by remaining in damp clothing, or in damp, cold rooms, until chilled through and through, or by standing or sitting still in the cold, after being a considerable time in a heated condition, whether from exercise or warm rooms. An attack of pneumonia always alarms the experienced physician. By knowing its causes, and wisely avoiding them, multitudes of valuable lives would be saved every year. Let every reader treasure these facts in his memory.

